# Farnesol and phosphorylation of the transcriptional regulator Efg1 affect *Candida albicans* white-opaque switching rates

**DOI:** 10.1371/journal.pone.0280233

**Published:** 2023-01-20

**Authors:** Lucas R. Brenes, Alexander D. Johnson, Matthew B. Lohse

**Affiliations:** 1 Department of Microbiology and Immunology, University of California, San Francisco, San Francisco, California, United States of America; 2 Department of Biochemistry and Biophysics, University of California, San Francisco, San Francisco, California, United States of America; Lebanese American University, LEBANON

## Abstract

*Candida albicans* is a normal member of the human microbiome and an opportunistic fungal pathogen. This species undergoes several morphological transitions, and here we consider white-opaque switching. In this switching program, *C*. *albicans* reversibly alternates between two cell types, named “white” and “opaque,” each of which is normally stable across thousands of cell divisions. Although switching under most conditions is stochastic and rare, certain environmental signals or genetic manipulations can dramatically increase the rate of switching. Here, we report the identification of two new inputs which affect white-to-opaque switching rates. The first, exposure to sub-micromolar concentrations of (E,E)-farnesol, reduces white-to-opaque switching by ten-fold or more. The second input, an inferred PKA phosphorylation of residue T208 on the transcriptional regulator Efg1, increases white-to-opaque switching ten-fold. Combining these and other environmental inputs results in a variety of different switching rates, indicating that a given rate represents the integration of multiple inputs.

## Introduction

*Candida albicans* is both a member of the human microbiome in healthy individuals and an opportunistic pathogen, causing diseases of varying severity, especially in individuals with a compromised immune system. These diseases can range from yeast infections and thrush to systemic bloodstream infections with fatality rates exceeding 40% [1–11]. *C*. *albicans* is also a polymorphic yeast that undergoes several distinct cell type switching programs. One of these is white-opaque switching, where *C*. *albicans* alternates between two cell types, named “white” and “opaque,” each of which exhibits distinct cellular and colony morphologies [12–19]. Switching between these two cell types is reversible and occurs without any chromosomal rearrangements or sequence changes [20]. Approximately one-sixth of the transcriptome is differentially regulated at least two-fold between white and opaque cells [21, 22], resulting in distinct metabolic preferences [21, 23, 24], capacities to mate [25], interactions with the innate immune system [26–30], and abilities to colonize and persist in different organs in the host [31]. The two cell types also respond to environmental cues in different ways; for example, the signals that induce white cells to form filaments are different from those that induce filamentation in opaque cells. Similarly, intermediate concentrations (roughly 40 μM) of the quorum sensing molecule (E,E)-farnesol are toxic to opaque cells while having little or no effect on white cells [32–35].

The stability of both cell types is a defining feature of the white-opaque switching program. Under standard laboratory conditions, switching between these two cell types occurs stochastically approximately once every 10^4^ cell divisions [36, 37]. Switching between the two cell types is controlled by a circuit with eight transcriptional regulators connected by interlocking feedback loops, with Efg1 and Wor1 being especially important for the establishment and maintenance of the white and opaque cell types respectively [38–50]. Switching rates between the two cell types are dependent on numerous signaling pathways, including the cAMP/protein kinase A pathway [23, 51–53], the Hog1 pathway [54–56], the Cek1 MAP kinase pathway [51, 55], and others [51, 57–65]. Environmental conditions that trigger these pathways also affect this switch, including elevated temperature and exposure to N-acetylglucosamine (GlcNAc) [12, 23, 36, 66]. Taken as a whole, the published literature demonstrate that white-opaque switching rates are highly responsive to many aspects of *C*. *albicans’* physiology.

Here, we report two additional inputs that affect the rates of white-opaque switching, namely sub-micromolar concentrations of (E,E)-farnesol and an inferred PKA phosphorylation of the transcriptional regulator Efg1. We describe how switching rates change in response to these two inputs, both by themselves and in combination with the environmental inputs GlcNAc and elevated temperature. Our results show that white-opaque switching rates reflect a complex combination of inputs, including the two described in this work, that is integrated to give a prescribed switching rate matched to a given set of conditions.

## Materials and methods

### Media and growth conditions

Unless otherwise noted, strains were grown and assays were performed on synthetic complete defined media plates containing yeast nitrogen base with 0.5% ammonium sulfate (6.7 g/L, BD #291940), amino acids (2 g/L), uridine (100 μg/mL), 2% glucose, and 2% agar (SCD+aa+Uri); 2% GlcNAc (Sigma #A3286) was added instead of glucose when relevant (SCGlcNAc+aa+Uri). Farnesol plates (e.g. SCD+aa+Uri+FOH) were made as follows. Following the autoclave step, the media was allowed to cool on a magnetic stir plate per our normal protocol. Before the media was poured into plates, the diluted farnesol solution (or equivalent volume of methanol for negative controls) was added to the media and stirred for an additional five to ten minutes. At this point, the media was poured into plates and allowed to cool and solidify overnight. Farnesol plates were always used in assays starting the day immediately after the day on which they were poured.

(E,E)-farnesol (*trans*,*trans*-3,7,11-Trimethyl-2,6,10-dodecatrien-1-ol, CAS number 106-28-5, Sigma #277541) requires special handling in order to remain effective. In brief, we have found that, even if initially aliquoted under anaerobic conditions, farnesol stock solutions stored in ambient air began to have reduced efficacy after one or two months. To avoid this issue, farnesol stock vials were only stored or worked with in an anaerobic chamber and all farnesol stock vials were discarded no more than two months after opening regardless of their frequency of usage. Dilutions, in methanol (Sigma #34860), were made in the anaerobic chamber on the day they were needed. To verify the effectiveness of the farnesol stocks used for each assay, we confirmed that a 10 μM farnesol solution diluted from that stock inhibited white cell filamentation in parallel with each farnesol assay. We do not expect farnesol efficacy to have changed during switching assays as the farnesol plates were exposed to air for a maximum of eight days from pouring to the end of said assays.

### Efg1 site location confirmation

The reported locations of the putative Cdc28:Hgc1 phosphorylation site at Threonine 179 [67] and putative PKA at phosphorylation site Threonine 206 [68, 69] do not correspond to Threonine residues in either the A or B alleles of Efg1 in the current *C*. *albicans* SC5314 Assembly 22 at the *Candida* Genome Database (CGD, candidagenome.org); instead, those residues are a glutamine and an arginine respectively. Based on the reported sequences of the regions immediately surrounding these two residues, we determined that they correspond to residues 181 (177-MQQP**T**PVQD-185) and 208 (204-RPRV**T**TTMW-212) in both Assembly 22 Efg1 alleles. To further verify this assignment, we aligned the original Efg1 sequence (GenBank accession Z32687) with both copies of the Assembly 19 and 22 SC5314 alleles as well as the WO-1 allele retrieved from CGD. This alignment revealed that the Z32687 sequence was missing, relative to the other strains, two alanine residues from a stretch of five alanines at residues 55–59; this accounts for the two amino acid difference between the initially reported (and still commonly listed) locations and the locations in the current genome assembly. We also note that one of the two Assembly 19 alleles and the WO-1 sequence differ by one amino acid (e.g. the putative phosphorylation sites are located at residues 180 and 207) due to the loss of one glutamine from a run of 11 glutamines at residues 85–95. Given the basis of the difference between the initially reported and current locations of the putative phosphorylation sites, we will name them based on the current genomic data and thus refer to them as Threonine 181 and Threonine 208, respectively.

### Strain construction

Lists of strains, plasmids, and oligonucleotides used in this study can be found in S1 File. The SC5314-derived *C*. *albicans* wild type white and opaque strains used in this study have been previously reported [46], in brief these are *HIS1* and *LEU2* addbacks to the SN152 **a**/α *his1 leu2 arg4* strain [70] that were then converted to the switching capable **a**/Δ background by deletion of the α copy of the Mating Type Like (*MTL*) locus using pJD1 [71].

The Efg1 T181 and T208 homozygous alanine and glutamic acid substitutions were constructed at the endogenous Efg1 locus in the wild type white strain utilizing the *SAT1* marker-based CRISPR protocol targeting *Candida maltosa LEU2* described by Nguyen and colleagues [72]. In brief, the 90bp-annealed donor DNA (dDNA) contains homology to the regions directly upstream and downstream of the targeted residue. Integration was confirmed by colony PCR and codon conversion was then confirmed by sequencing. For the *czf1* deletion construction in the wild type and Efg1 T208E strain backgrounds, the 90bp-annealed donor DNA (dDNA) contains homology to the regions directly upstream and downstream of the *CZF1* ORF. Each dDNA homology arm consisted of 44bp and the two arms were separated by a two base pair GG insert to create a potential gRNA site. Gene deletion was confirmed by colony PCR reactions verifying loss of the *CZF1* ORF. In all cases, after confirming the presence of the desired edit, the Cas9 ORF-gRNA-*SAT1* cassette was recycled by plating on Leu/His/Arg dropout plates and selecting for recombination events with an intact *CmLEU2* ORF. We selected against both leucine and histidine in order to avoid potential histidine auxotrophies arising during the recombination process as both *CmLEU2* and *CdHIS1* are present at the *CaLEU2* locus in this strain background. Consistent with previous reports [73], our *czf1* deletion strain is insensitive to farnesol in regards to inhibition of filamentation.

Two independent isolates were constructed for both the T208A and T208E mutations. Given the lack of a white-opaque switching phenotype, only one isolate was constructed for the T181A and T181E mutations. Although only single isolates were constructed for the *czf1* deletion and *czf1* deletion + Efg1 T208E strains, these two strains are independent of each other as *czf1* was deleted independently in the wild type and Efg1 T208E backgrounds to make the respective strains.

### White-opaque switching assays

White-to-opaque and opaque-to-white switching assays followed previously reported protocols [25, 43, 64]. In brief, strains were allowed to recover from glycerol stocks for seven days on SCD+aa+Uri plates at 25°C. After seven days, five colonies per strain that lacked visible sectors of the other cell type were resuspended in water and plated at a concentration of approximately 100 colonies/plate. The number of plates used varied based on the expected switching rates and the number of conditions being tested, ranging from as few as five to as many as fifteen. Unless otherwise noted, assays were performed on SCD+aa+Uri plates and plates were incubated for seven days at 25°C before scoring. For the elevated temperature (37°C) assays, plates were incubated for three days and scored on the third day. Three phenotypes were noted: (1) the number of sectored colonies, (2) the number of fully switched colonies, and (3) the total number of colonies. The overall switching frequency (called “Overall Switching Frequency” in tables) was calculated as 100 * (number of sectored colonies + number of fully switched colonies) / total number of colonies. The full colony switching events (called “Full Colony Switching Events” in tables) was calculated as 100 * number of fully switched colonies / total number of colonies. Full Colony Switching Events represent cases where switching events occurred either prior to or immediately after a cell was plated which resulted in an entire colony exhibiting the derived rather than parental phenotype (e.g. a full opaque colony rather than a white colony with one or more opaque sectors). The overall switching rate includes both of these events as well as events that occurred after a cell was plated which give rise to one or more sectors. Large increases of the Full Colony Switching Event rate in white-to-opaque switching assays are typically associated with mutations and/or environmental conditions that strongly drive white-to-opaque switching (e.g. ectopic overexpression of *WOR1* or growth on GlcNAc with 5% CO_2_) as very high rates of switching are needed in order to produce full opaque colonies as opposed to white colonies with numerous opaque sectors. If no switching events were observed, the rate is reported as less than 100 / total number of colonies. Depending on the basal switching rate in a particular assay, we generally consider three- to five-fold or greater changes in switching rates to be significant. The number of plates used are included in the legend of each table. Unless otherwise noted, each table contains data from one replicate of an experiment conducted in parallel on the same day and all plates with a given strain were seeded from the same resuspension. Key experiments were repeated on at least two separate days; data reported in a table are from a representative repeat of a given experiment. Two independent isolates were screened for the T208E and T208A strains, the data presented in any given table reflects a single isolate. As the T181A and T181E strains did not have an interesting switching phenotype, only one isolate was screened for these strains. Only one isolate was screened for both the *czf1* deletion and *czf1* deletion + Efg1 T208E strains however, as noted above, these two strains are independent of each other (being constructed in the wild type and Efg1 T208E backgrounds respectively) and similar trends were observed in each case.

## Results

### Submicromolar farnesol exposure inhibits white-to-opaque switching

White and opaque cells have different responses to the quorum sensing molecule (E,E)-farnesol; white cells produce farnesol and tolerate farnesol concentrations of at least 250 μM with minimal effects on growth while farnesol concentrations as low as 40 μM result in widespread opaque cell death [34, 35]. Farnesol is also known to affect the *C*. *albicans* yeast-to-hyphal transition: exposure to 1 μM farnesol results in 50% inhibition of white cell filamentation [74]. Based on farnesol’s known effects, we hypothesized that farnesol concentrations might affect white-opaque switching. To test this hypothesis, we performed white-to-opaque switching assays in the presence of either 0.1 μM or 1 μM farnesol. We found that, under standard lab conditions (glucose at 25°C), 0.1 μM farnesol decreased wild type white-to-opaque switching ten-fold with 1 μM farnesol inhibiting switching even further (Table 1). Thus, a farnesol concentration roughly one-tenth of that needed to affect the yeast-to-hyphal transition inhibited white-to-opaque switching by ten-fold. Neither 0.1 μM nor 1 μM farnesol affected the reverse switching rate, that is switching of opaque cells to white cells (Table 1). The unidirectional nature of farnesol’s effect is similar to that of many of the gene deletions and environmental signals that affect white-opaque switching, and is consistent with the apparent independence of the mechanisms for the establishment and maintenance of the opaque cell type [23, 64, 65].

**Table 1:** Farnesol switching frequencies. Wild type white-to-opaque and opaque-to-white switching frequencies in the presence of 0 μM (methanol-only control), 0.1μM, and 1μM farnesol scored after seven days growth on SCD+aa+Uri plates at 25°C. Ten plates were scored per condition.

White-to-Opaque Switching				
Strain	Farnesol (μM)	Overall Switching Frequency (%)	Full Colony Switching Events (%)	n
Wild Type	0.0	1.39	< 0.13	794
Wild Type	0.1	0.12	< 0.12	823
Wild Type	1.0	< 0.12	< 0.12	867
Opaque-to-White Switching				
Strain	Farnesol (μM)	Overall Switching Frequency (%)	Full Colony Switching Events (%)	n
Wild Type	0.0	27.67	24.24	524
Wild Type	0.1	28.07	26.12	513
Wild Type	1.0	20.54	18.43	521

### PKA phosphorylation of Efg1 affects white-opaque switching rates

The transcriptional regulator Efg1 is a core regulator of white-opaque switching but also affects biofilm formation and the yeast-to-hyphal transition. In the context of white-opaque switching, Efg1 is crucial to the establishment and maintenance of the white cell type [38, 43, 68, 69, 75–77]. Efg1 is known to be phosphorylated based on the observation that it runs as two or three distinct bands on denaturing protein gels which collapse to a single band when treated with phosphatase; subsequent phosphoproteome studies have detected as many as six distinct phosphorylation events [52, 69, 78–83]. It has also been noted, based on changes in the abundance of different protein species in two-dimensional gels, that the phosphorylation state of Efg1 is different between yeast and hyphal cells [80]. Based on sequence analyses, Efg1 has been predicted to be phosphorylated by Cdc28:Hgc1 at Threonine 181 (177-MQQP**T**PVQD-185) and by PKA at Threonine 208 (204-RPRV**T**TTMW-212). As described in the Methods, these residues are frequently identified as T179 and T206 but correspond to T181 and T208 in the current SC5314 genome Assembly 22. The putative phosphorylation state of these sites, as inferred by mutations to these residues, affects both expression of Efg1 and Efg1’s ability to regulate the yeast-to-hyphal transition [67, 69, 81, 84]. It is important to note that phosphorylation of T208 has not been detected in three independent phosphoproteome studies, suggesting that it might be a rare modification [52, 82, 83]. Because multiple components of the PKA pathway upstream of Efg1 (e.g. *GPA2*, *TPK1*, *TPK2*, *BCY1*) have also been linked to the regulation of white-opaque switching [23, 51–53], we hypothesized that the phosphorylation state of Efg1 at either T181 or T208 might have an impact on white-opaque switching rates.

To test this hypothesis, we independently mutated T181 and T208 to glutamic acid (T181E, T208E), to mimic constitutive phosphorylation, and alanine (T181A, T208A), to create a non-phosphorylated state. We then determined whether any of these mutations affected white-to-opaque or opaque-to-white switching under our standard laboratory conditions (glucose at 25°C). The Efg1 T181E, T181A, and T208A mutants had minimal effects on switching in either direction and we will not consider them further (Table 2 and S1 Table). The T208E mutant, on the other hand, increased white-to-opaque switching more than ten-fold and reduced opaque-to-white switching between three- and ten-fold (Table 2 and S1 Table). We consider the implications of these results for white-opaque switching in the discussion.

**Table 2:** Efg1 phosphomimetic switching frequencies. White-to-opaque and opaque-to-white switching frequencies for wild type and T181 and T208 Efg1 phosphomimetic strains scored after seven days growth on SCD+aa+Uri plates at 25°C. Six plates were scored per condition.

White-to-Opaque Switching			
Strain	Overall Switching Frequency (%)	Full Colony Switching Events (%)	n
Wild Type	1.68	< 0.21	477
Efg1 T208E	21.65	< 0.22	462
Efg1 T208A	3.43	< 0.21	467
Efg1 T181E	1.66	< 0.21	482
Efg1 T181A	2.96	< 0.15	676
Opaque-to-White Switching			
Strain	Overall Switching Frequency (%)	Full Colony Switching Events (%)	n
Wild Type	20.83	19.79	288
Efg1 T208E	1.89	1.18	424
Efg1 T208A	13.82	13.39	463
Efg1 T181E	14.75	12.84	366
Efg1 T181A	12.01	11.17	358

### *C*. *albicans* switching rates reflect the influence of multiple independent inputs

Having examined how these inputs affected switching rates in isolation, we next considered the effect of combining them with each other and with two additional known inputs. We first considered the effects of farnesol (0.1 μM, 1 μM, or 10 μM), the Efg1 T208E phosphomimetic mutation, and GlcNAc (in different pairwise combinations) on white-to-opaque switching (Table 3 and S2 Table). When we combined an input that increased white-to-opaque switching (either the T208E phosphomimetic or GlcNAc) with an input that decreased white-to-opaque switching (farnesol), we observed an intermediate switching rate that was lower than either the T208E phosphomimetic or GlcNAc by themselves but was higher than that observed for farnesol by itself (Table 3 and S2 Table). When we combined the two inputs that increased white-to-opaque switching (the T208E phosphomimetic and GlcNAc), we observed an additive effect where the switching rate was higher than either input by itself (two- to three-fold higher than the T208E phosphomimetic on glucose and 14- to 20-fold higher rate than the wild type strain on GlcNAc) (Table 3). We note that this high rate of switching was dependent on the presence of the T208E phosphomimetic mutation, neither the wild type nor the T208A mutant displayed a similar phenotype on GlcNAc (Table 3 and S3 Table). Much of this increase in the switching rate stems from a 40- to 160-fold increase in early switching events giving rise to fully opaque colonies (rather than white colonies with one or more opaque sectors) (Table 3). This behavior is similar to the previously reported phenotype associated with the *bcy1* deletion (which results in constitutive activation of the PKA kinase) on GlcNAc [53].

**Table 3:** White-to-opaque farnesol, Efg1 phosphomimetic, and GlcNAc switching frequencies. White-to-opaque switching frequencies for the wild type and Efg1 T208E strains scored after seven days growth at 25°C on SCD+aa+Uri or SCGlcNAc+aa+Uri plates in the presence of 0 μM (methanol-only control), 0.1μM, 1μM, or 10 μM farnesol. Five plates were scored per condition.

White-to-Opaque Switching					
Strain	Media	Farnesol (μM)	Overall Switching Frequency (%)	Full Colony Switching Events (%)	n
Wild Type	SCD+aa+Uri	0.0	1.39	0.20	502
Wild Type	SCD+aa+Uri	0.1	0.21	< 0.21	484
Wild Type	SCD+aa+Uri	1.0	< 0.20	< 0.20	501
Wild Type	SCD+aa+Uri	10.0	< 0.22	< 0.22	452
Wild Type	SCGlcNAc+aa+Uri	0.0	2.92	0.19	514
Wild Type	SCGlcNAc+aa+Uri	0.1	0.90	0.45	444
Wild Type	SCGlcNAc+aa+Uri	1.0	1.16	0.23	431
Wild Type	SCGlcNAc+aa+Uri	10.0	0.82	< 0.21	485
Efg1 T208E	SCD+aa+Uri	0.0	19.73	0.53	375
Efg1 T208E	SCD+aa+Uri	0.1	2.35	< 0.29	341
Efg1 T208E	SCD+aa+Uri	1.0	2.25	< 0.28	355
Efg1 T208E	SCD+aa+Uri	10.0	0.93	< 0.31	322
Efg1 T208E	SCGlcNAc+aa+Uri	0.0	41.54	22.55	337
Efg1 T208E	SCGlcNAc+aa+Uri	0.1	2.44	0.54	369
Efg1 T208E	SCGlcNAc+aa+Uri	1.0	3.67	0.31	327
Efg1 T208E	SCGlcNAc+aa+Uri	10.0	1.69	< 0.28	354

Given the additive effect of the T208E phosphomimetic and GlcNAc inputs on white-to-opaque switching rates, we next evaluated how these combined inputs interacted with the effect of temperature. In glucose media at 25°C, opaque cells are stable through many cell divisions, but when the temperature is raised to 37°C, opaque cells switch *en masse* to white cells. Likewise, growth at 37°C normally prevents white-to-opaque switching [12, 36]. We observed occasional white-to-opaque switching events at 37°C with the T208E phosphomimetic mutant on GlcNAc, albeit at a much lower rate than was observed for either of those inputs by themselves at 25°C (Table 4). This result suggests that the T208E mutation can override, at least to some extent, the dramatic effect caused by an increase in temperature.

**Table 4:** 37°C switching assays. White-to-opaque switching frequencies for the wild type and T208E Efg1 phosphomimetic strains grown for either three days at 37°C or seven days at 25°C on either SCD+aa+Uri or SCGlcNAc+aa+Uri plates. Five plates were scored per condition.

White-to-Opaque Switching					
Strain	Media	Temperature (°C)	Overall Switching Frequency (%)	Full Colony Switching Events (%)	n
Wild Type	SCD+aa+Uri	25	1.98	< 0.28	353
Wild Type	SCD+aa+Uri	37	< 0.25	< 0.25	406
Wild Type	SCGlcNAc+aa+Uri	25	4.30	< 0.29	349
Wild Type	SCGlcNAc+aa+Uri	37	< 0.28	< 0.28	351
Efg1 T208E	SCD+aa+Uri	25	23.32	0.26	386
Efg1 T208E	SCD+aa+Uri	37	< 0.32	< 0.32	310
Efg1 T208E	SCGlcNAc+aa+Uri	25	64.13	41.67	276
Efg1 T208E	SCGlcNAc+aa+Uri	37	0.69	0.69	290

### Farnesol affects white-opaque switching in a *CZF1*-independent manner

Farnesol’s inhibition of the yeast-to-hyphal transition is dependent on the transcriptional regulator Czf1: a *czf1* deletion strain fails to be inhibited [73]. Czf1 is also important for the establishment of the opaque cell type and for proper expression of a subset of the opaque cell transcriptional program [43, 44, 46]. We tested whether Czf1 was needed for farnesol’s effect on white-to-opaque switching and we found that the *czf1* deletion strain’s white-to-opaque switching rates were still reduced when exposed to 1 μM farnesol (Table 5). We observed this result for the *czf1* deletion strain when GlcNAc was the carbon source and in a *czf1* deletion combined with the Efg1 T208E mutation when either glucose or GlcNAc were the carbon source (Table 5). These results suggest that although farnesol’s inhibition of filamentation depends on Czf1, its effect on white-to-opaque switching does not.

**Table 5:** *czf1* deletion farnesol white-to-opaque switching assays. White-to-opaque switching frequencies for the wild type, *czf1* deletion, T208E Efg1 phosphomimetic, and *czf1* deletion plus T208E Efg1 phosphomimetic strains grown for seven days on either SCD+aa+Uri or SCGlcNAc+aa+Uri plates. Five plates were scored per condition for the T208E Efg1 phosphomimetic and the *czf1* deletion plus T208E Efg1 phosphomimetic strains, fifteen plates were scored per condition for the wild type and *czf1* deletion strains.

White-to-Opaque Switching					
Strain	Media	Farnesol (μM)	Overall Switching Frequency (%)	Full Colony Switching Events (%)	n
Wild Type	SCGlcNAc+aa+Uri	0.0	1.83	0.08	1313
Wild Type	SCGlcNAc+aa+Uri	1.0	0.55	< 0.08	1269
Δ/Δ*czf1*	SCGlcNAc+aa+Uri	0.0	0.51	0.17	1167
Δ/Δ*czf1*	SCGlcNAc+aa+Uri	1.0	0.08	< 0.08	1277
Efg1 T208E	SCD+aa+Uri	0.0	19.09	< 0.30	330
Efg1 T208E	SCD+aa+Uri	1.0	1.10	< 0.27	365
Δ/Δ*czf1* + Efg1 T208E	SCD+aa+Uri	0.0	1.54	< 0.26	389
Δ/Δ*czf1* + Efg1 T208E	SCD+aa+Uri	1.0	< 0.24	< 0.24	410
Efg1 T208E	SCGlcNAc+aa+Uri	0.0	37.85	19.95	391
Efg1 T208E	SCGlcNAc+aa+Uri	1.0	1.36	< 0.27	367
Δ/Δ*czf1* + Efg1 T208E	SCGlcNAc+aa+Uri	0.0	14.62	10.21	431
Δ/Δ*czf1* + Efg1 T208E	SCGlcNAc+aa+Uri	1.0	0.70	< 0.23	430

## Discussion

Here, we report that the putative PKA phosphorylation of residue T208 on the transcriptional regulator Efg1 increases white-to-opaque switching rates and that exposure to sub-micromolar concentrations of (E,E)-farnesol decreases white-to-opaque switching rates in *C*. *albicans*. Farnesol did not affect the reverse transition, opaque-to-white switching rates (at 25°C on glucose), a unidirectional effect that had been observed previously for other mutations and environmental conditions [23, 64, 65]; these results further underscore the independence of the mechanisms for the establishment versus the maintenance of the opaque cell type. Unlike the yeast-to-hyphal transition, where farnesol’s negative effects are mediated by Czf1, we found that Czf1 was not required for farnesol to affect white-to-opaque switching, suggesting that the two effects are mediated, at least in part, through different signaling pathways.

Because farnesol can act as a quorum sensing molecule, our results suggest that population density could affect the frequency of white-to-opaque switching. Saturated cultures of white cells have been reported to have farnesol concentrations ranging from 13 to 59 μM [85], well above the concentrations that eliminated white-to-opaque switching in our study (at 25°C on glucose). This result suggests that switching to the opaque cell type would be favored in relatively dilute cultures.

Our results also provide insight into the signaling pathways that influence the regulation of white-opaque switching and how the white-opaque regulatory circuit incorporates information from multiple pathways. The phenotype of the Efg1 T208E phosphomimetic resembles that of the constitutively active PKA mutant (the *bcy1* deletion) [53] and we hypothesize that the cAMP/PKA pathway’s effects on white-opaque switching are at least partially mediated through phosphorylation of Efg1 at residue T208. Our demonstration that an Efg1 phosphorylation mimic at this residue affects white-opaque switching suggests a link between the previous observations that (1) Efg1 regulates white-opaque switching, (2) that deletion of cAMP/PKA signaling pathway genes affects white-opaque switching rates, and (3) the inference that Efg1 is phosphorylated by PKA at T208 [23, 38, 43, 52, 53, 67–69, 76, 81, 84, 86]. Still, it is important to note that PKA-related phosphorylation of T208 has yet to be experimentally verified and was not detected in three independent phosphoproteome studies. We note, however. that these three studies were conducted under conditions (e.g. 37°C) optimized for hyphal growth rather than white-opaque switching [52, 82, 83], and it is possible that the steady-state of T208 phosphorylation is low. It remains to be definitively determined whether Efg1 is indeed phosphorylated at the T208 site.

Assuming the PKA-T208 phosphorylation hypothesis is correct, it suggests that PKA signaling through this residue affects switching rates but, under normal laboratory conditions (room temperature, glucose, ambient air), has only a moderate effect and does not eliminate the effects of other signals (e.g. farnesol) which in many cases may be dominant. The T208E switching results suggest that putative phosphorylation of T208 promotes the establishment and/or maintenance of the opaque cell type; however, the results of the T208A mutation suggest that phosphorylation of this residue is not required. Such a result is not unprecedented: overexpression of Wor3 increased white-to-opaque switching but its deletion had little or no effect [48]. The results observed when we combined inputs to white-opaque switching were not always “additive”, indicating that the process that integrates the different inputs is complex. For example, the additive effects of the Efg1 T208E phosphomimetic and GlcNAc, compared to the intermediate effects of T208E and farnesol, illustrate the complex nature of the integration process and suggest that some inputs may be relatively independent. For example, our results, in which GlcNAc and the T208E phosphomimetic have an additive effect, are consistent with recent reports that GlcNAc does not signal through the cAMP/PKA pathway [87, 88]. Likewise, farnesol is known to affect Cek1-, Hog1-, and Chk1-mediated signaling in addition to its effects on the cAMP/PKA pathway [89–91]. As such, the intermediate switching effect seen for the combination of farnesol and the T208E phosphomimetic could reflect farnesol’s effects on one or more of these other pathways, at least two of which are known to affect white-opaque switching [51, 54, 56].

In summary, our results indicate that *C*. *albicans* integrates multiple inputs to determine the final white-opaque switching rate and that multiple weaker inputs can override the effect of a normally strong input such as growth at 37°C. The abnormally large control regions upstream of the core regulator genes of white-opaque switching and the numerous mutations affecting switching rates suggest that *C*. *albicans* integrates a great deal of information to control the balance between the two cell types.

## Supporting information

S1 Table Efg1 phosphomimetic switching frequencies. White-to-opaque and opaque-to-white switching frequencies for wild type and independent T208E and T208A isolates scored after seven days growth on SCD+aa+Uri plates at 25°C. Seven plates were scored per condition.
(XLSX)

S2 Table White-to-opaque Efg1 phosphomimetic farnesol and GlcNAc switching frequencies. White-to-opaque switching frequencies for an independent Efg1 T208E isolate scored after seven days growth at 25°C on SCD+aa+Uri or SCGlcNAc+aa+Uri plates in the presence of 0 μM (methanol-only control) or 1μM farnesol. Ten plates were scored per condition.
(XLSX)

S3 Table White-to-opaque Efg1 T208A and GlcNAc switching frequencies. White-to-opaque switching frequencies for the wild type and Efg1 T208A strains scored after seven days growth at 25°C on SCD+aa+Uri or SCGlcNAc+aa+Uri plates. Five plates were scored per condition.
(XLSX)

S1 File List of strains, plasmids, and oligonucleotides used in this study. (XLSX)
